# Loss of T follicular regulatory cell–derived IL-1R2 augments germinal center reactions via increased IL-1

**DOI:** 10.1172/jci.insight.174005

**Published:** 2024-02-08

**Authors:** Katerina Pyrillou, Melanie Humphry, Lauren A. Kitt, Amanda Rodgers, Meritxell Nus, Martin R. Bennett, Kenneth G.C. Smith, Paul A. Lyons, Ziad Mallat, Murray C.H. Clarke

**Affiliations:** 1 Section of CardioRespiratory Medicine, Heart and Lung Research Institute, and; 2 Cambridge Institute of Therapeutic Immunology and Infectious Disease, Jeffrey Cheah Biomedical Centre, University of Cambridge, Cambridge Biomedical Campus, Cambridge, United Kingdom.

**Keywords:** Immunology, Inflammation, Adaptive immunity

## Abstract

Inappropriate immune activity is key in the pathogenesis of multiple diseases, and it is typically driven by excess inflammation and/or autoimmunity. IL-1 is often the effector owing to its powerful role in both innate and adaptive immunity, and, thus, it is tightly controlled at multiple levels. IL-1R2 antagonizes IL-1, but effects of losing this regulation are unknown. We found that IL-1R2 resolves inflammation by rapidly scavenging free IL-1. Specific IL-1R2 loss in germinal center (GC) T follicular regulatory (Tfr) cells increased the GC response after a first, but not booster, immunization, with an increase in T follicular helper (Tfh) cells, GC B cells, and antigen-specific antibodies, which was reversed upon IL-1 blockade. However, IL-1 signaling is not obligate for GC reactions, as WT and *Il1r1*^–/–^ mice showed equivalent phenotypes, suggesting that GC IL-1 is normally restrained by IL-1R2. Fascinatingly, germline *Il1r2*^–/–^ mice did not show this phenotype, but conditional *Il1r2* deletion in adulthood recapitulated it, implying that compensation during development counteracts IL-1R2 loss. Finally, patients with ulcerative colitis or Crohn’s disease had lower serum IL-1R2. All together, we show that IL-1R2 controls important aspects of innate and adaptive immunity and that IL-1R2 level may contribute to human disease propensity and/or progression.

## Abstract 2

IL-1 promotes antibody production after germinal center T and B cell interaction, while IL-1 receptor 2 from regulatory cells inhibits IL-1 and reduces antibody generation.

## Introduction

IL-1 is evolutionarily ancient (1), and it exerts effect on both innate and adaptive processes. Within humans, the IL-1 family comprises 11 ligands and 10 receptors that both promote and inhibit inflammation. IL-1α and IL-1β are the principal ligands that bind to the type 1 IL-1 receptor (IL-1R1) and recruit IL-1 receptor accessory protein (IL-1RAP) to enable interaction with the signaling adapter MyD88 (2). Following a phosphosignaling cascade NF-κB is activated, leading to the predominantly studied proinflammatory effects of IL-1 (2) that include cytokine secretion, endothelial cell activation, upregulation of adhesion molecules and MHC/costimulatory molecules, and increased vascular permeability (3). These powerful actions are countered by a receptor antagonist (IL-1RA) that inhibits IL-1 binding to IL-1R1, and a decoy receptor (IL-1R2).

The affinity of IL-1R2 and IL-1R1 for IL-1 is similar, but IL-1R2 lacks the intracellular TIR domain needed to signal, and it is the first identified example of a decoy receptor (4). IL-1R2 is suggested to inhibit IL-1 signaling by competing with IL-1R1 for IL-1 at the cell surface, binding of IL-1 to soluble IL-1R2, and competing IL-1RAcP away from IL-1R1; additionally, an intracellular form of IL-1R2 prevents IL-1α activation (5, 6). Soluble IL-1R2 is generated by alternative splicing (7) or metalloprotease-like dependent shedding from the cell surface (8), which can be induced by LPS, TNF-α, and PMA (8). Similarly, expression of IL-1R2 transcripts is induced by glucocorticoids, IL-4, and IL-13 and decreased by LPS and IFN-γ (8). IL-1R2 can also undergo internalization upon binding to IL-1, with the receptors returned to the surface after removal of ligand (9). IL-1R2 expression has traditionally been ascribed to B cells and monocytes (8), but gene expression profiling (Immgen) shows Langerhans and neutrophils to be the highest expressers. However, there are scarce data examining the mechanism and action of IL-1R2 within a physiological process or disease setting in vivo.

The role of IL-1 in the adaptive immune system is less studied, but reported effects include enhanced survival and expansion of T cells, Th17 cell differentiation, and effector T cell proliferation in the presence of Tregs (10–12). Importantly, emerging evidence suggests IL-1 signaling may control B cell function and antibody production in germinal centers (GCs). GCs represent sites within secondary lymphoid organs for sophisticated antibody responses, such as isotype switching, affinity maturation, and generation of plasma cells and memory B cells. Within GCs, follicular helper T (Tfh) cells drive B cell maturation to plasma cells, while follicular regulatory T (Tfr) cells suppress responses. However, unlike Tregs, Tfr cells do not express CD25 and thus cannot bind IL-2 (13). In contrast, Tfh cells express IL-1R1 while Tfr cells express the IL-1 antagonists IL-1R2 and IL-1RA. Thus, IL-1 signaling by Tfh cells in vitro drives IL-4 and IL-21 production that matures B cells, while Tfr cells may counteract this with IL-1R2 and IL-1RA. However, if this IL-1 axis regulates GC reactions and antibody production after immunization in vivo is not known.

Although IL-1 is clearly central in the pathogenesis of multiple diseases, most preclinical studies utilize models with total loss of IL-1 signaling, often via global loss of IL-1R1 or IL-1α/β. In contrast, very few reports have examined the effects of more moderate alterations in IL-1 signaling. Interestingly, loss of the antagonist IL-1RA leads to polyarthropathy (14) and arterial inflammation (15) under the steady state, suggesting a basal level of IL-1 activity must continually be suppressed to maintain homeostasis. Indeed, the marvel of the immune system is how it delicately balances positive and negative regulation to maintain a “Goldilocks level” of response large enough to remove an insult, but without collateral tissue damage and/or loss of tolerance. Again, how IL-1R2 contributes to immune homeostasis is unknown.

We found that IL-1R2 is important in resolving inflammation by scavenging free IL-1 and that membrane-associated IL-1R2 has little effect on a given cell’s sensitivity to IL-1. Loss of IL-1R2 in Tfr cells altered the adaptive response after immunization, with more T follicular (Tfol) cells and GC B cells and increased antigen-specific IgG antibody titers, along with activation and polarization of CD4^+^ and CD8^+^ T cells, which was reversed upon anakinra treatment to block IL-1. However, IL-1 signaling was not required for GC reactions, as WT and *Il1r1*^–/–^ mice showed identical responses, suggesting that IL-1R2 normally restrains IL-1 in the GC. Unexpectedly, germline R2^–/–^ mice did not show this phenotype, but conditional IL-1R2 loss in adulthood fully recapitulated it, implying that compensation within the IL-1 family during development can counteract against loss of IL-1R2. In addition, individuals with ulcerative colitis (UC) or Crohn’s disease had lower serum levels of IL-1R2. Thus, IL-1R2 controls critical aspects of both innate and adaptive immunity, and IL-1R2 level may be an important predictor of human disease.

## Results

### Cre-mediated excision of Il1r2 exon 3 results in loss of IL-1R2 protein.

**Figure 1:**
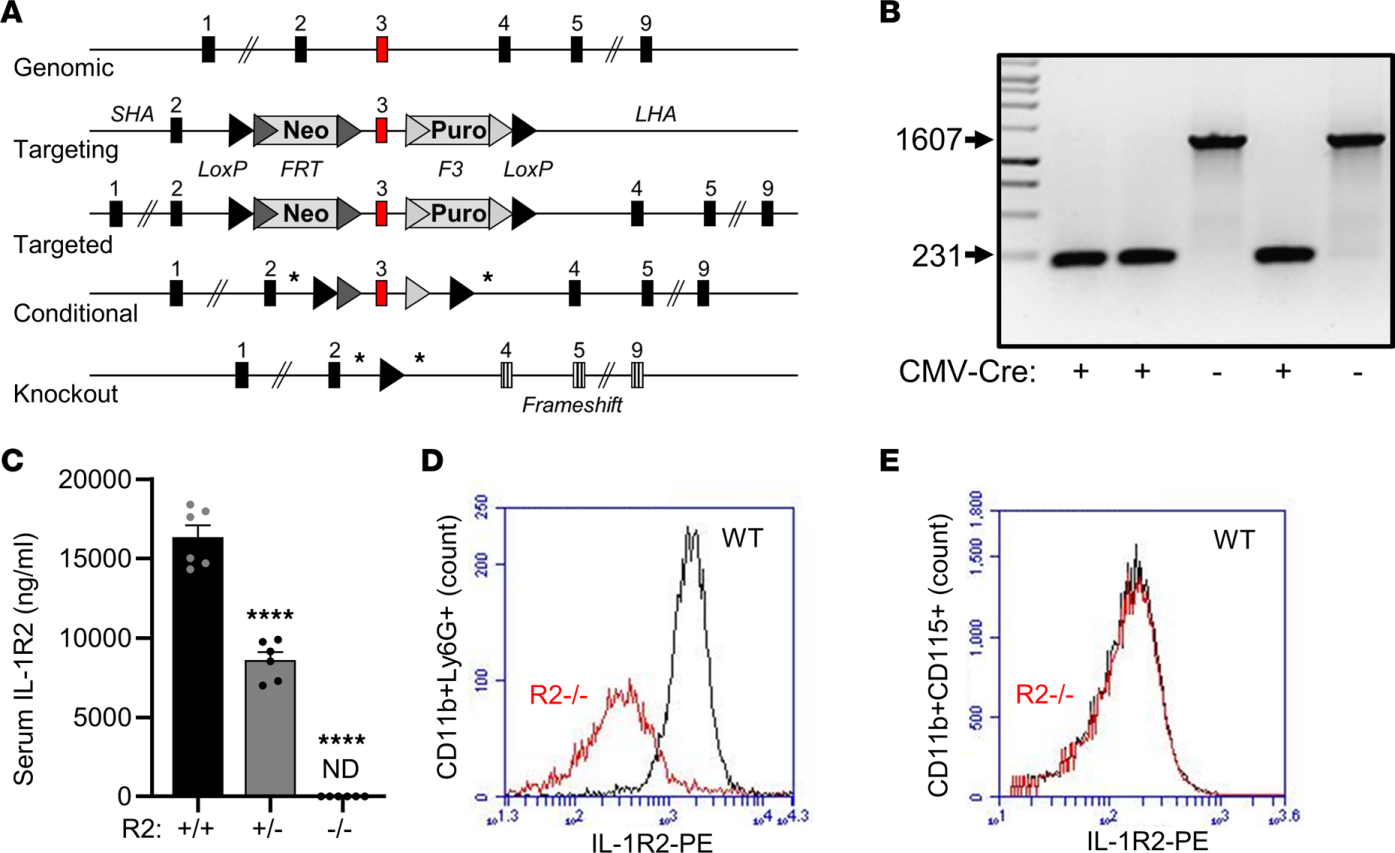
Cre-mediated excision of *Il1r2* exon 3 results in loss of IL-1R2 protein. (A) Targeting strategy for generation of *Il1r2* floxed mice. SHA, short homology arm; LHA, long homology arm. The asterisks indicate the position of genotyping primers. (**B**) Separated PCR products showing CMV-Cre–mediated removal of approximately 1.4 kb of *Il1r2* containing exon 3. Values shown are in base pairs (BP). (**C**) ELISA data for serum IL-1R2 in female mice with IL-1R2 genotypes as indicated. (**D** and **E**) Flow cytometric analysis for IL-1R2 and lineage markers on whole blood from IL-1R2^+^/^+^ (WT) and IL-1R2^–/–^ (R2^–/–^) mice, showing IL-1R2 level on the surface of neutrophils (**D**) and monocyte/macrophages (**E**). Data are shown as the mean ± SEM of *n* = 6 (**C**) and are representative of *n* = 4 (**D** and **E**). ****P ≤ 0.0001, *t* test.

IL-1R2 is the first identified example of a decoy receptor and is proposed to reduce inflammation by inhibiting IL-1 signaling. However, proof of mechanism of action in vivo is lacking. Thus, we generated mice deficient in IL-1R2 by CMV-Cre–mediated removal of exon 3 from *Il1r2* (Figure 1A), followed by selective crossing to remove CMV-Cre. These mice lacked exon 3 in the genome by PCR (Figure 1B), and sequencing confirmed the correct recombination that left only a LoxP site in the genome (data not shown). Notably, ELISA showed an approximately 50% reduction in soluble IL-1R2 in the sera of *Il1r2*^+/–^ mice compared with that of *Il1r2*^+/+^ (WT) mice and no detectable serum IL-1R2 in *Il1r2*^–/–^ knockout (R2^–/–^) mice (Figure 1C). In addition, flow cytometry of whole blood showed a measurable loss of IL-1R2 on neutrophils from R2^–/–^ mice compared with those from WT mice (Figure 1D), but no difference on monocytes/macrophages (Figure 1E), which express lower levels of surface IL-1R2. R2^–/–^ mice were viable and displayed no gross morphological or phenotypic difference (data not shown). These data show that deletion of *Il1r2* exon 3 results in loss of IL-1R2 protein.

### IL-1R2–deficient mice show a prolonged inflammatory response due to uncleared IL-1.

**Figure 2:**
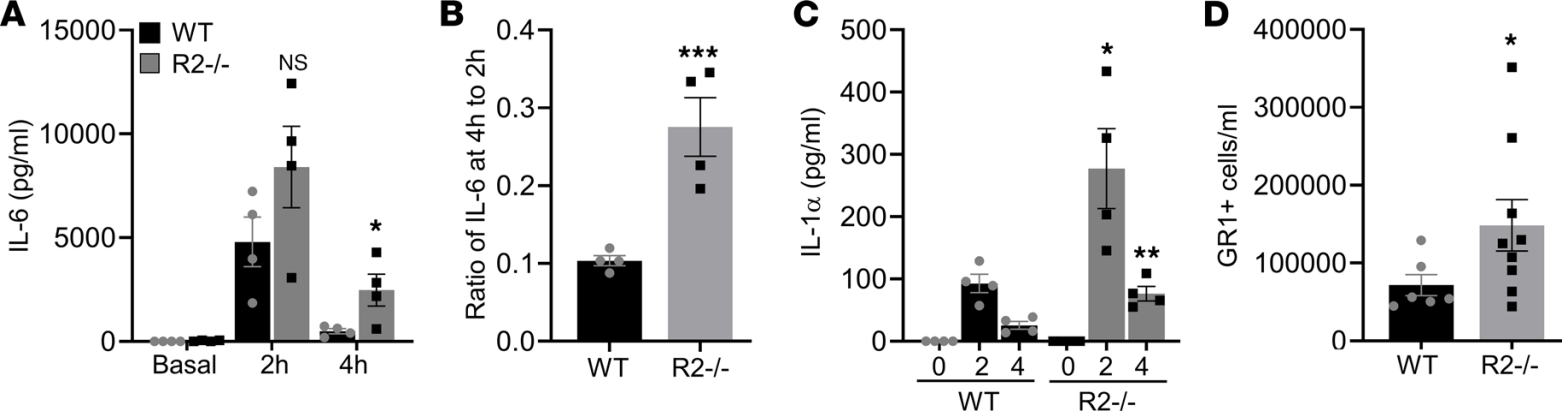
IL-1R2–deficient mice show a prolonged inflammatory response due to uncleared IL-1. (**A**) ELISA data for serum IL-6 at the indicated times after intravenous injection of IL-1α into IL-1R2^+/+^ (WT) and IL-1R2^–/–^ (R2^–/–^) mice. (**B**) Ratio of serum IL-6 at 4 hours to that at 2 hours after intravenous injection of IL-1α. (**C**) ELISA data for serum IL-1α at the indicated times after intravenous injection of IL-1α. (**D**) Flow cytometry for the number of GR1^+^ cells recruited into the peritoneum 6 hours after i.p. injection of IL-1α. Data are shown as the mean ± SEM of *n* = 4 (**A**–**C**) and *n* ≥ 6 (**D**). *P ≤ 0.05, **P ≤ 0.02, ***P ≤ 0.005, *t* test.

To investigate whether deletion of *Il1r2* affects IL-1–driven processes in vivo, we intravenously injected IL-1α and collected sera over time to measure induced cytokine level. No significant difference in the level of IL-6 between groups was found after 2 hours (Figure 2A). However, after 4 hours sera IL-6 levels were higher in R2^–/–^ mice and nearly undetectable in WT mice (Figure 2A). Indeed, comparing the ratio of IL-6 at 4 hours to that at 2 hours revealed a substantial prolonging of the inflammatory reaction in R2^–/–^ mice (Figure 2B), suggesting IL-1–driven inflammation does not resolve as swiftly without IL-1R2. To test if this was due to a lack of IL-1 scavenging by the high concentration of IL-1R2 in serum (16), we directly measured the serum level of the injected human IL-1α over time. This showed markedly increased levels of IL-1α at both time points in R2^–/–^ mice compared with those in WT mice (Figure 2C), supporting reduced clearance of IL-1α from the circulation. Using a sterile peritonitis model, we examined the effect of IL-1R2 loss without the high level of IL-1R2 in the circulation, which showed increased neutrophil recruitment in response to IL-1α in R2^–/–^ mice (Figure 2D). Together, these data show that IL-1R2 limits inflammation and scavenges free IL-1 and, thus, that R2^–/–^ mice have increased IL-1 signaling.

### T follicular regulatory cell–derived IL-1R2 restrains GCs and antibody production.

**Figure 3:**
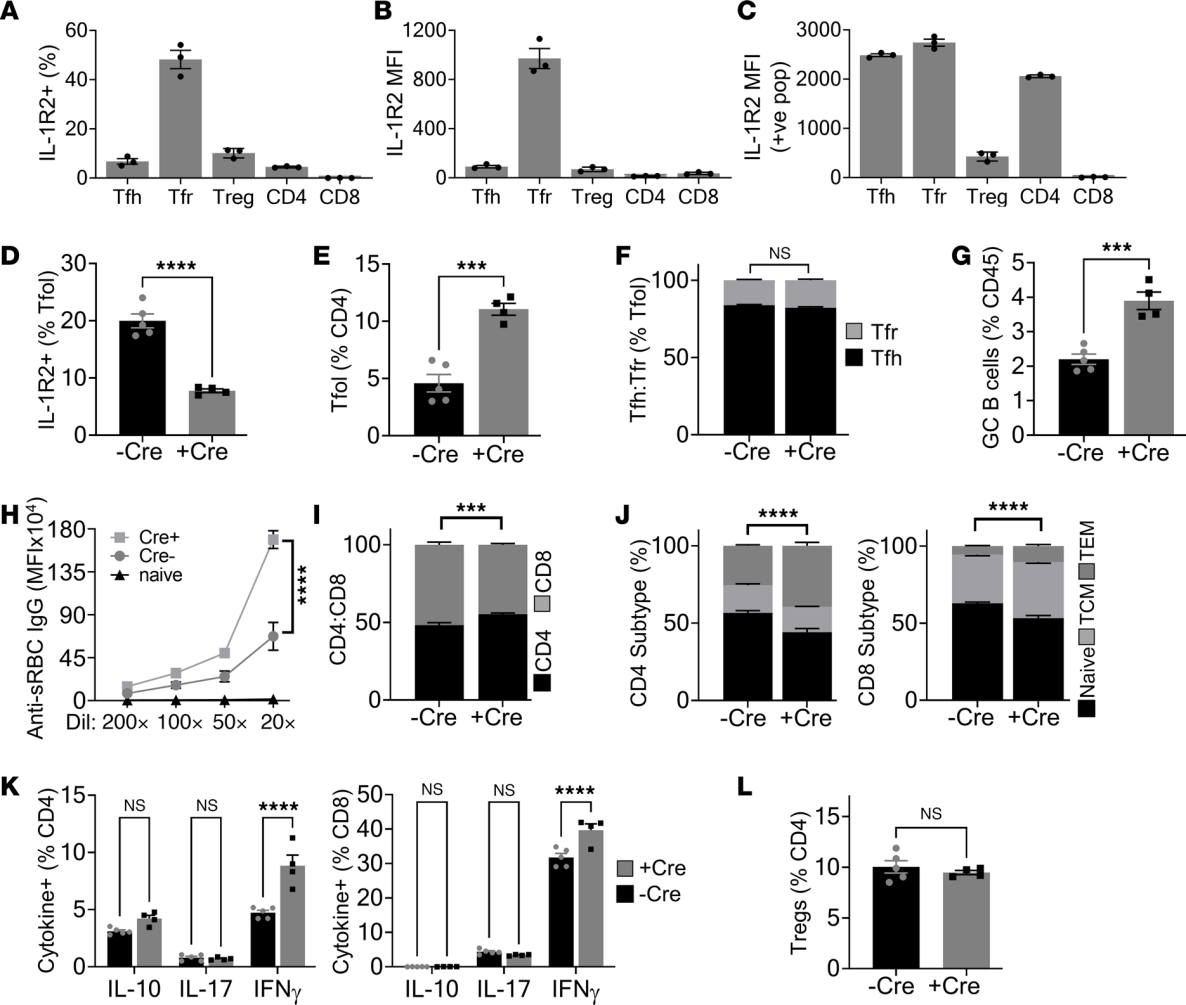
Loss of IL-1R2 in Tfr cells increases the GC response after immunization. Flow cytometry for IL-1R2 on WT splenic T follicular helper (Tfh) cells, T follicular regulatory (Tfr) cells, Tregs, and CD4^+^ or CD8^+^ T cells, showing the percentage of population positive for IL-1R2 (**A**) and median fluorescent intensity (MFI) of the whole population (**B**) and only those positive for IL-1R2 (**C**). Presented values for WT mice have percentage and MFI seen in IL-1R2^–/–^ mice subtracted. (**D–L**) *Il1r2*^fl/fl^ ± Foxp3-Cre-ER^T2^ (Cre) littermate mice were all tamoxifen treated and immunized with sheep red blood cells (sRBC); spleens were immunophenotyped 8 days later. (**D**–**G**) Flow cytometry for IL-1R2 on splenic T follicular (Tfol) cells (**D**), Tfol cells (**E**), the ratio of Tfh cells to Tfr cells (**F**), and germinal center (GC) B cells **(G**) in the genotypes indicated. (**H**) Flow cytometry for binding of serum anti-sRBC IgG antibodies to sRBCs. (**I** and **J**) Flow cytometry for the splenic CD4^+^/CD8^+^ T cell ratio (**I**) and CD4^+^/CD8^+^ T cell subtype (**J**) in the genotypes indicated. TCM, central memory; TEM, effector memory. (**K**) Intracellular cytokine staining in splenic CD4^+^/CD8^+^ T cells activated with PMA/ionomycin. (**L**) Flow cytometry for splenic Tregs. Data are shown as the mean ± SEM; *n* = 3 individual mice (**A**–**C**) or *n* = 5 individual -Cre and 4 individual +Cre mice (**D**–**L**) representative of ≥3 repeats. ***P ≤ 0.001, ****P ≤ 0.0001, *t* test and ANOVA.

**Figure 4:**
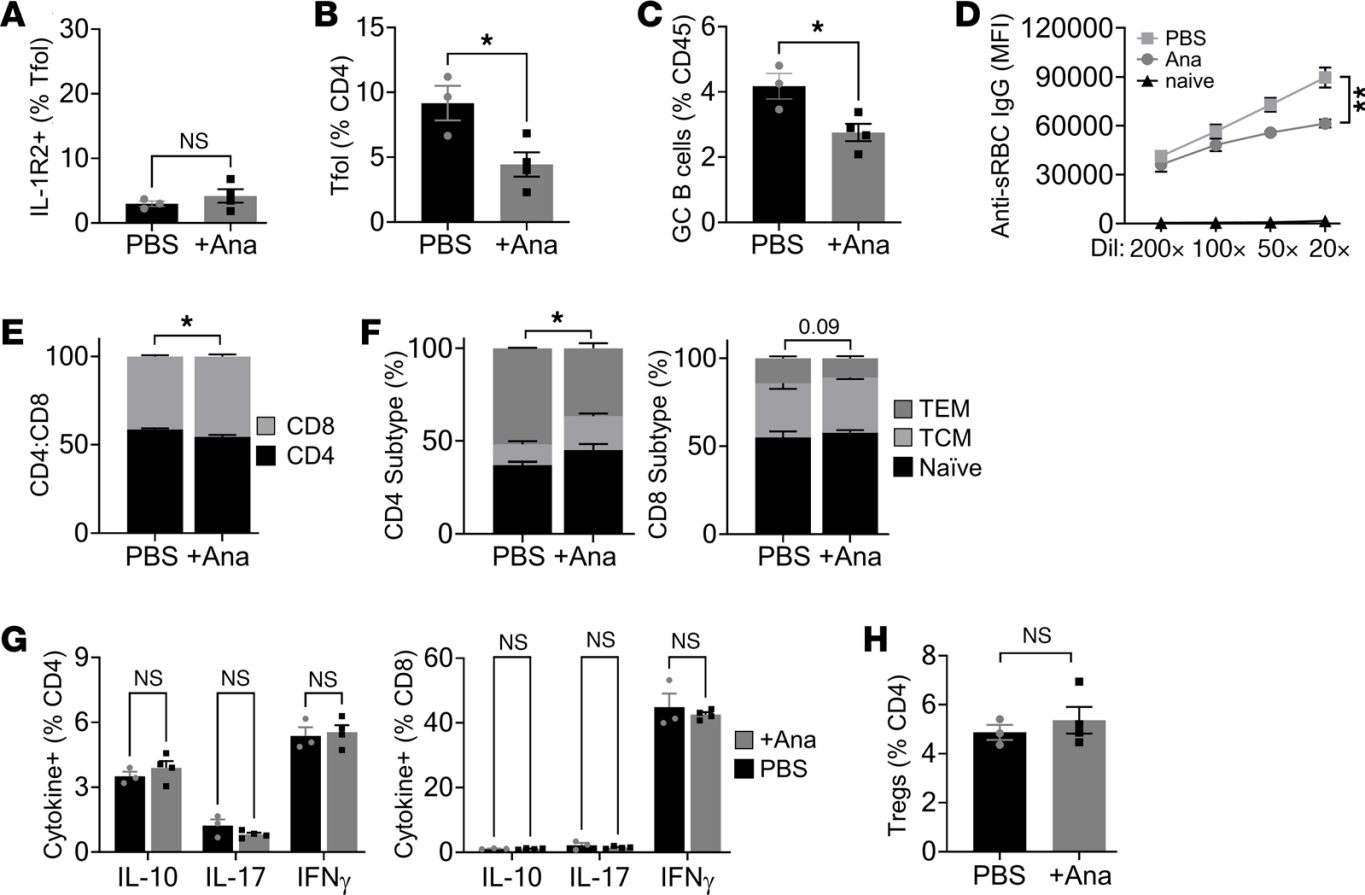
Anakinra blocks the heightened GC response in mice lacking Tfr IL-1R2. *Il1r2*^fl/fl^/Foxp3-Cre-ER^T2^ littermate mice were all tamoxifen treated, immunized with sheep red blood cells (sRBC), along with concurrent PBS or Anakinra (Ana) treatment until spleen immunophenotyping 8 days later. (**A**–**C**) Flow cytometry for IL-1R2 on splenic T follicular (Tfol) cells (**A**), Tfol cells (**B**), and germinal center (GC) B cells (**C**) in the genotypes indicated. (**D**) Flow cytometry for binding of serum anti-sRBC IgG antibodies to sRBCs. (**E** and **F**) Flow cytometry for splenic CD4^+^/CD8^+^ T cell ratio (**E**) and CD4^+^/CD8^+^ T cell subtype (**F**) in the genotypes indicated. TCM, central memory; TEM, effector memory**.** (**G**) Intracellular cytokine staining in splenic CD4^+^/CD8^+^ T cells activated with PMA/ionomycin. (**H**) Flow cytometry for splenic Tregs. Data are shown as the mean ± SEM; *n* = 3 individual mice (PBS) or *n* = 4 individual mice (+Ana). *P ≤ 0.05, **P ≤ 0.01, *t* test and ANOVA.

As loss of IL-1R2 can increase availability of IL-1 (Figure 2), we examined processes known to be IL-1 dependent. GC reactions are driven by interaction between T follicular helper (Tfh) cells and B cells, with T follicular regulatory (Tfr) cells providing negative regulation (17). Tfr cells are suggested to dampen GC reactions by producing IL-1R2 that antagonizes IL-1 activation of Tfh cells (13), but if this occurs in vivo and any consequence of interfering with this regulation is unknown. We produced *Il1r2*^fl/fl^*/Foxp3*Cre^ERT2+^ mice to enable conditional deletion of IL-1R2 in Tfr cells. As Tfr cells expressed the highest level of IL-1R2 (Figure 3, A–C, and Supplemental Figure 1; supplemental material available online with this article; https://doi.org/10.1172/jci.insight.174005DS1), while Tregs expressed IL-1R2 at a lower frequency and level (Figure 3, A–C, and Supplemental Figure 1) (in keeping with previous reports; ref. 13) and do not accumulate in the GC, this supports that the main target of *Foxp3*-directed IL-1R2 deletion in the GC is the Tfr cell. Without tamoxifen, splenocyte cDNA only generated the *Il1r2*^fl/fl^ amplicon, but tamoxifen via diet lead to generation of the *Il1r2*^–/–^ amplicon and a loss of the *Il1r2*^fl/fl^ band (Supplemental Figure 2). Importantly, MACS sorting for Tregs/Tfr cells resulted in near-complete loss of the *Il1r2*^fl/fl^ amplicon and generation of the *Il1r2*^–/–^ band (Supplemental Figure 2). In addition, approximately 20% of Tfol cells (CD4/CXCR5/PD-1^+^) expressed IL-1R2, and tamoxifen treatment resulted in a large decrease in this signal (Figure 3D). Together, Tfr cells are the major Tfol cell expressing IL-1R2, and tamoxifen induces *Foxp3*-directed deletion of IL-1R2 in Tfr cells. Challenging this model with the classic sheep red blood cell (sRBC) immunization resulted in more Tfol cells (Figure 3E) but no change in the ratio of Tfh to Tfr cells (Figure 3F), expanded GCs (Figure 3G), as evidenced by increased GL7/B220^+^ B cells, and much higher titers of serum anti-sRBC IgG (Figure 3H) in mice lacking IL-1R2 in Tfr cells. GCs showed normal gross morphology between groups, with no increase in follicle size (Supplemental Figure 3A), but more GL7^+^ area per follicle was seen with Tfr IL-1R2 loss (Supplemental Figure 3B), in keeping with the flow cytometry data. In addition, *Il1r2*^fl/fl^*/Foxp3*Cre^ERT2+^ mice showed increased numbers of CD4^+^ T cells (Figure 3I) and altered ratios of naive and effector/central memory CD4^+^ and CD8^+^ T cells (Figure 3J) in the spleens; splenocytes stimulated ex vivo with PMA/ionomycin showed increased IFN-γ^+^ CD4^+^ and CD8^+^ T cells (Figure 3K), compared with mice lacking *Foxp3*Cre^ERT2^. However, splenic Treg level was not altered with IL-1R2 deletion (Figure 3L). Near identical results were found when OVA/Alum was used for immunization (Supplemental Figure 4), suggesting a general effect of IL-1R2 loss on GC responses. Most importantly, administering Anakinra (IL-1RA) to block both IL-1α and IL-1β reversed the increased responses seen in *Il1r2*^fl/fl^*/Foxp3*Cre^ERT2+^ mice (Figure 4), showing that the action of IL-1R2 loss is mediated via IL-1. All together, this shows that deficiency of IL-1R2 in Tfr cells augments GC reactions and antibody output after immunization and that this occurs due to increased IL-1 signaling.

### Tfr IL-1R2 deficiency does not increase GC reactions after a booster immunization.

As IL-1R1 signaling can amplify GC responses, we investigated if loss of IL-1R2 on Tfr cells would further heighten GC reactions and antibody production after a second booster immunization of sRBCs. Interestingly, *Il1r2*^fl/fl^*/Foxp3*Cre^ERT2+^ mice still showed increased Tfol cells and CD4^+^ T cells, altered ratios of CD4^+^/CD8^+^ subtypes, and increased IFN-γ expression in CD4^+^/CD8^+^ T cells (Supplemental Figure 5, A–G), as seen before (Figure 3, D–L). However, the titer of serum anti-sRBC IgG was no different between *Il1r2*^fl/fl^*/Foxp3*Cre^ERT2+^ and *Il1r2*^fl/fl^ mice (Supplemental Figure 5H), with levels in *Il1r2*^fl/fl^*/Foxp3*Cre^ERT2+^ mice no higher than those seen after a single immunization (Figure 3H) but anti-sRBC IgG levels in *Il1r2*^fl/fl^ mice boosted to the same level as those witnessed in *Il1r2*^fl/fl^*/Foxp3*Cre^ERT2+^ mice (Supplemental Figure 5H). Interestingly, IL-1R2 on Tfol cells in boosted *Il1r2*^fl/fl^ mice dropped to an equivalent level (~10%) (Supplemental Figure 5A) as that seen in mice lacking Tfr IL-1R2 (Figure 3D and Figure 4A), suggesting that a second immunization may intrinsically lower GC IL-1R2. Together, without IL-1R2 on Tfr cells, a higher GC output can be reached after a single immunization, while a subsequent booster generates similar responses with IL-1R2 present.

### IL-1R1 signaling is not essential for induction of the GC response after immunization.

**Figure 5:**
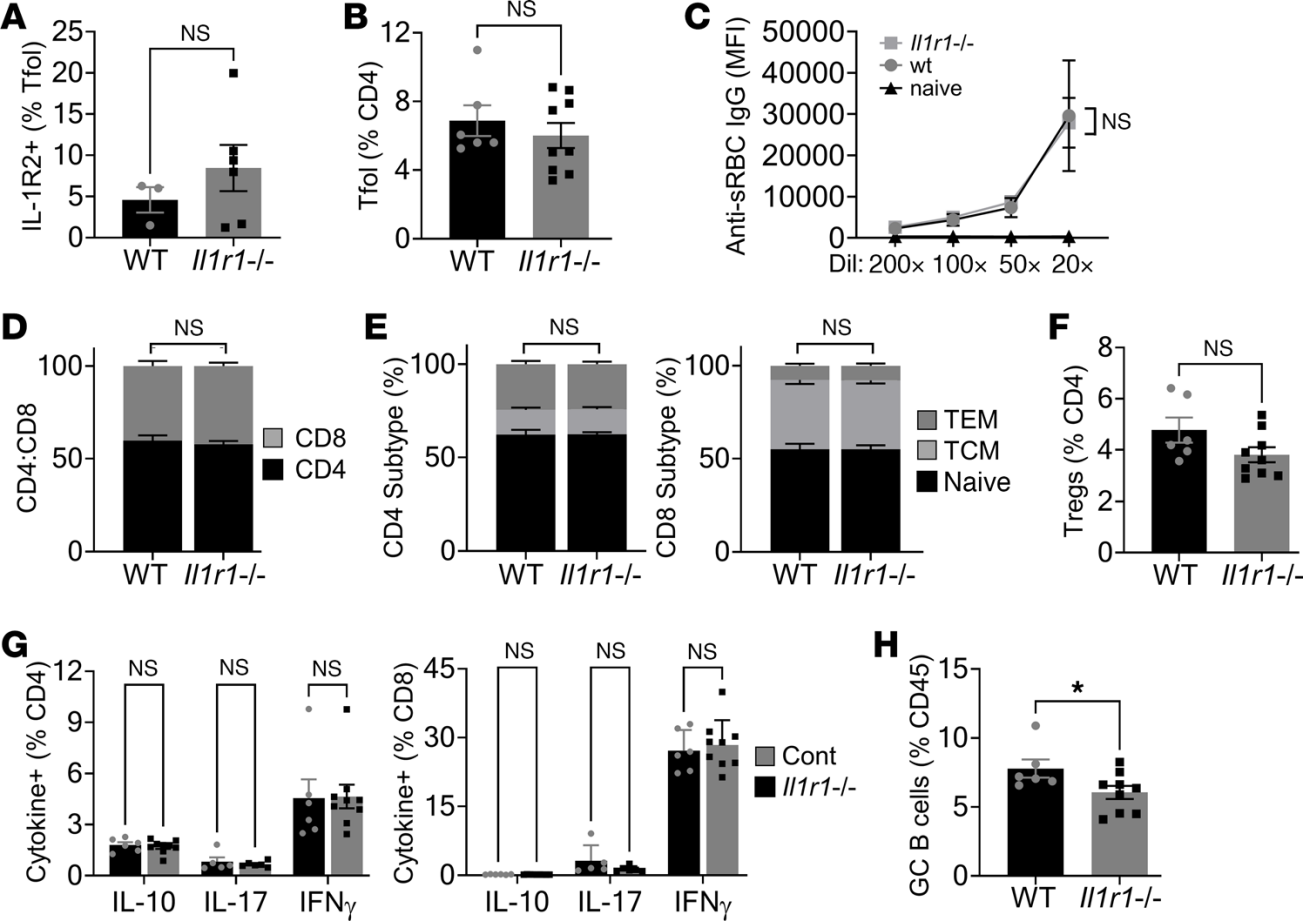
IL-1R1 signaling is not essential for induction of the GC response after immunization. *Il1r1^–/–^ andIl1r1^+/+^* (WT) littermate mice were immunized with sheep red blood cells (sRBC) and spleens were immunophenotyped 8 days later. (**A** and **B**) Flow cytometry for IL-1R2 on splenic T follicular (Tfol) cells (**A**) and Tfol cells (**B**) in the genotypes indicated. (**C**) Flow cytometry for binding of serum anti-sRBC IgG antibodies to sRBCs. (**D**–**F**) Flow cytometry for splenic CD4^+^/CD8^+^ T cell ratio (**D**), CD4^+^/CD8^+^ T cell subtype (**E**), and Tregs (**F**) in the genotypes indicated. TCM, central memory; TEM, effector memory. (**G**) Intracellular cytokine staining in splenic CD4^+^/CD8^+^ T cells activated with PMA/ionomycin. (**H**) Flow cytometry for splenic germinal center (GC) B cells (**H**). *n* = 3 individual WT mice or *n* = 6 individual *Il1r1^–/–^* mice (**A**) and *n* = 6 individual WT mice or *n* = 9 individual *Il1r1^–/–^* mice (**B**–**H**). *P ≤ 0.05, *t* test and ANOVA.

As the major action of IL-1R2 is to bind IL-1, increased GC responses with IL-1R2–deficient Tfr cells suggest that IL-1R1 signaling may normally be required for GC reactions. To investigate this, we immunized littermate *Il1r1*^+/+^ and *Il1r1*^–/–^ mice with sRBC and again performed immunophenotyping of the spleens. Surprisingly, loss of IL-1R1 had very little effect on the GCs, with no difference in the number of Tfol cells (Figure 5A), Tfol IL-1R2 expression (Figure 5B), titers of serum anti-sRBC IgG (Figure 5C), T cell numbers (Figure 5D), subtype (Figure 5E), Tregs (Figure 5F), or activation status (Figure 5G) and only a marginal decrease in the number of GC B cells (Figure 5H). This shows that IL-1 is not obligatory for a productive GC reaction and suggests that IL-1R2 normally suppresses IL-1 in the GC, with the enhancing effects of IL-1 only apparent when IL-1R2 level is compromised.

### GC reactions are only increased with temporal loss of IL-1R2 in adulthood.

**Figure 6:**
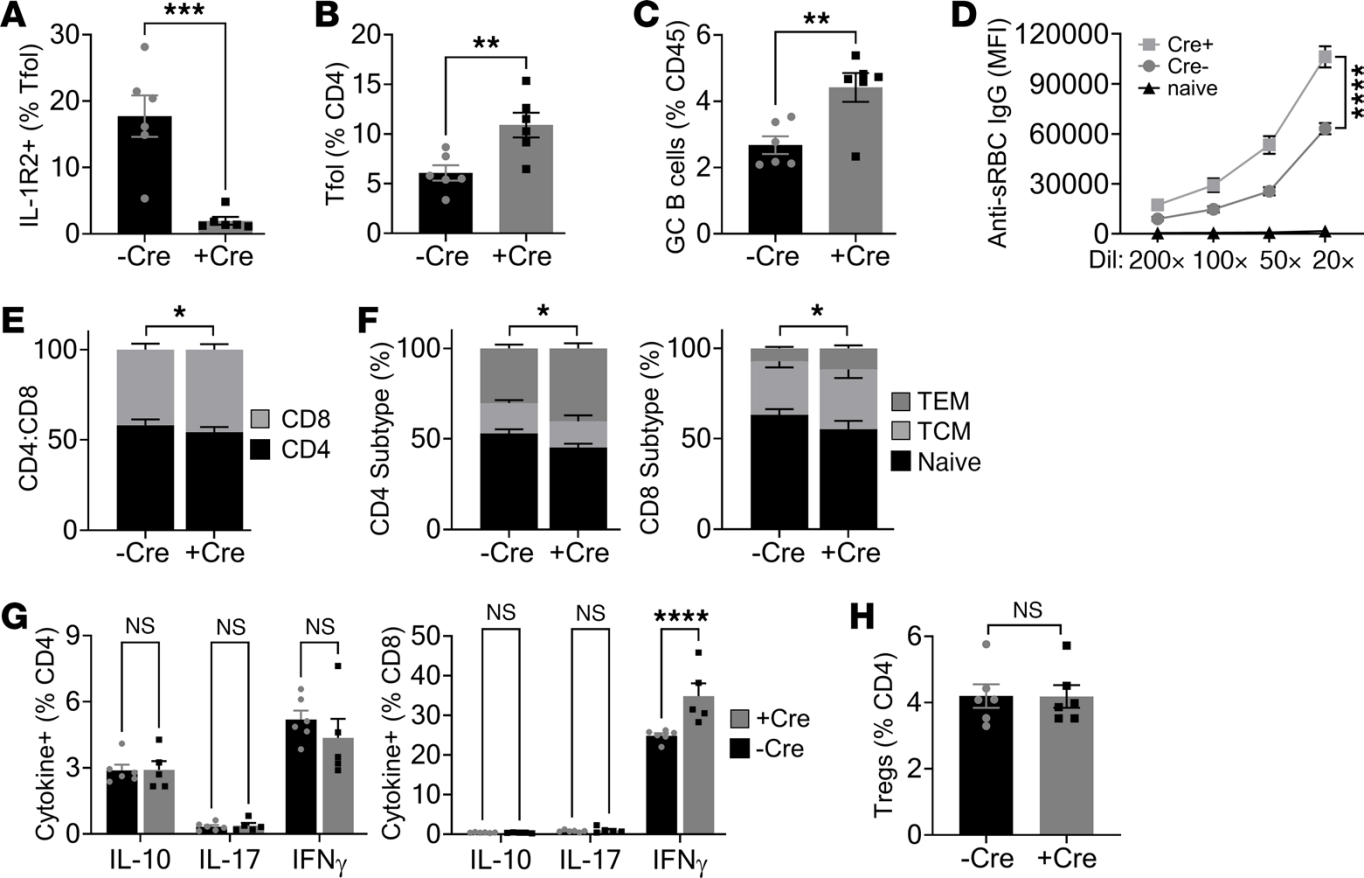
Conditional global loss of IL-1R2 in adulthood increases the GC response after immunization. *Il1r2*^fl/fl^ ± Rosa26-Cre-ER^T2^ (Cre) littermate mice were all tamoxifen treated and immunized with sheep red blood cells (sRBC); spleens were immunophenotyped 8 days later. (**A**–**C**) Flow cytometry for IL-1R2 on splenic T follicular (Tfol) cells (**A**), Tfol cells (**B**), and germinal center (GC) B cells (**C**) in the genotypes indicated. (**D**) Flow cytometry for binding of serum anti-sRBC IgG antibodies to sRBCs. (**E** and **F**) Flow cytometry for splenic CD4^+^/CD8^+^ T cell ratio (**E**) and CD4^+^/CD8^+^ T cell subtype (**F**) in the genotypes indicated. TCM, central memory; TEM, effector memory. (**G**) Intracellular cytokine staining in splenic CD4^+^/CD8^+^ T cells activated with PMA/ionomycin. (**H**) Flow cytometry for splenic Tregs. Data are shown as the mean ± SEM; *n* = 6 individual mice (both -Cre and +Cre). *P ≤ 0.05, **P ≤ 0.01, ***P ≤ 0.001, ****P ≤ 0.0001, *t* test and ANOVA.

As global IL-1R2^–/–^ mice show altered IL-1–driven responses (Figure 2) and total loss of the high levels of soluble IL-1R2 in sera (Figure 1C), we tested if they also showed different GC dynamics. Unexpectedly, IL-1R2^–/–^ mice only had a weak phenotype, comprising increased CD4^+^ T cell numbers, altered CD8 subtype ratio, and increased IFN-γ^+^ CD8^+^ T cells (Supplemental Figure 6, A–C). However, IL-1R2^–/–^ mice showed no changes to Treg, Tfol cell, or GC B cell number or anti-sRBC IgG titers (Supplemental Figure 6, D–G), compared with littermate IL-1R2^+^/^+^ mice. We reasoned that the anomaly between Tfr-specific and global IL-1R2 deletion could be caused by IL-1R2 somehow having a “dual function,” such as opposing effects within the GC versus systemic, or more likely that it was caused by the temporal deletion of IL-1R2 via Cre/Lox in adulthood that was important. To test the second possibility, we generated *Il1r2*^fl/fl^/Rosa26-Cre^ERT2^ mice to allow conditional IL-1R2 deletion upon tamoxifen treatment. Fascinatingly, globally deleting IL-1R2 in adulthood gave a near-identical phenotype (Figure 6) after sRBC immunization to that seen with IL-1R2 deletion in Tfr cells (Figure 3, D–L), with the only tangible difference being more IL-1R2 deletion in the Tfol cells (Figure 6A), likely due to deletion of IL-1R2 on Tfh and Tfr cells and a more efficient Cre. All together, this suggests a complicated system in which IL-1R2 normally restrains GC reactions but that absence of IL-1R2 from conception leads to some form of compensation that mitigates against its absence in adulthood. Indeed, similar functional compensation has been seen in other gene families (18), but the mechanism remains poorly understood.

### Individuals with inflammatory bowel disease have lower serum IL-1R2.

**Figure 7:**
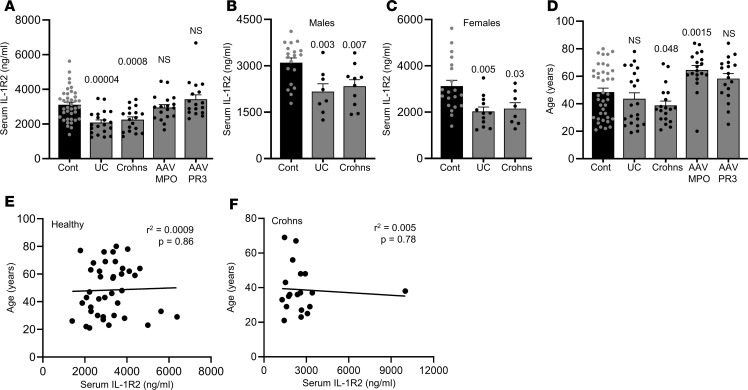
Individuals with inflammatory bowel disease have lower serum IL-1R2. (**A**–**C**) ELISA data for serum IL-1R2 levels in humans with ulcerative colitis (UC), Crohn’s disease (Crohns), ANCA-associated vasculitis MPO^+^ (AAV-MPO), and AAV proteinase 3^+^ (AAV-PR3) or individuals acting as healthy controls (Cont) displayed for both male and female patients (**A**), and male (**B**) or female (**C**) patients only. (**D**) Age of individuals with conditions as indicated. (**E** and **F**) Scatter plot of serum IL-1R2 versus age for individuals acting as healthy controls (**E**) and patients with Crohn’s disease (**F**) showing no correlation. Data are shown as the median ± SEM of *n* = 40 (Cont), 20 (UC), 19 (Crohns, AAV-MPO), 18 AAV-PR3). *P* values are stated in the figure (ANOVA and linear regression).

If IL-1R2 can control GC reactions and/or alter CD4^+^/CD8^+^ T cell function, lower levels of IL-1R2 could lead to autoimmune and/or inflammatory disease in humans. To initially investigate this, we collected samples from patients with UC, Crohn’s disease, and antineutrophil cytoplasmic antibody–associated (ANCA-associated) vasculitis (AAV) positive for myeloperoxidase (AAV-MPO) or proteinase 3 (AAV-PR3) — diseases with well-documented autoimmune and autoinflammatory components. Testing serum IL-1R2 level revealed substantially decreased IL-1R2 in patients with UC and Crohn’s disease but no difference in patients with either form of AAV (Figure 7A), compared with individuals acting as healthy controls. Lower serum IL-1R2 was witnessed in both male (Figure 7B) and female (Figure 7C) patients with UC and Crohn’s disease. Although there was a statistically significant age difference between patients with Crohn’s disease and patients with AAV-MPO and individuals acting as healthy controls (Figure 7D), sera IL-1R2 levels did not correlate to age in either healthy control (Figure 7E) or Crohn’s disease (Figure 7F) populations, suggesting that age was not a confounding factor for IL-1R2 level. Interestingly, GWAS have identified common *IL1R2* variants as a key risk factor for UC (19) and Crohn’s disease (20). All together, these initial data reveal that lower levels of serum IL-1R2 are associated with human disease.

## Discussion

The immune system constantly maintains a delicate balance to produce a response that sufficiently resolves an insult with minimum collateral damage to the host. Thus, all immune processes are controlled by feedback mechanisms that promote or inhibit responses. IL-1 activates crucial innate and adaptive processes, and extensive study has revealed multiple levels of control. Most investigations utilize global knockout of IL-1R1 or IL-1, resulting in total loss of signaling. However, most inflammatory and/or autoimmune diseases are driven by altered production of IL-1 within the affected tissue, with very few reports investigating more modest alterations in IL-1 activity on immune responses.

Here, we show that the decoy receptor IL-1R2 is central in resolving inflammation by scavenging free IL-1. Deletion of IL-1R2 in Tfr cells lead to an altered adaptive response after immunization, with increased Tfol cells, GC B cells, and antigen-specific IgG antibody titers, along with marked activation and polarization of CD4^+^ and CD8^+^ T cells, which was reversed upon IL-1 blockade. Interestingly, IL-1 signaling was not obligate for GC reactions, as WT and *Il1r1*^–/–^ mice showed identical responses, and boosting WT mice increased antibody titers to levels seen in mice lacking IL-1R2 in Tfr cells, suggesting that IL-1 is normally restrained in the GC by IL-1R2. Fascinatingly, germline R2^–/–^ mice did not show this immune phenotype, but global deletion of IL-1R2 in adulthood fully recapitulated it, implying that compensation within the IL-1 family can counteract against chronic loss of IL-1R2 but not acute conditional loss. Finally, patients with UC or Crohn’s disease have lower serum IL-1R2. All together, this work shows that IL-1R2 is important for controlling aspects of both innate and adaptive immunity and that levels of IL-1R2 may be a predictor of human disease propensity and/or progression.

IL-1R2 is the first identified decoy receptor, and it is proposed to bind IL-1 and prevent signaling via IL-1R1. IL-1R2 is found on the surface of many cell types (8), as a cytosolic intracellular form (5, 6) and as a soluble form at high concentrations in the circulation (16). Pragmatically, as IL-1R2 is only known to bind IL-1, any phenotype upon IL-1R2 loss is highly likely due to more IL-1 signaling. Previous work using R2^–/–^ mice show increased susceptibility to K/BxN- and collagen-induced arthritis (21, 22), with particularly high IL-1R2 expression noted on neutrophils (23). However, T cell responses and antibody production to collagen were normal (21), in keeping with our findings in global R2^–/–^ mice (Supplemental Figure 6). R2^–/–^ mice also show increased infarct size and myeloid cell recruitment after coronary artery ligation and reperfusion (24). However, no additional work has reported the effect of IL-1R2 loss on adaptive immune processes. IL-1 drives Th17 differentiation and, therefore, is central in many autoimmune diseases. However, IL-1 is also vital for effective generation of Th1 cells, where signaling through IL-1R1 on naive or effector CD4^+^ T cells is required to overcome the inhibitory signals from Tregs (12), and it is also required for the generation of functionally competent memory CD4^+^ T cells (12). IL-1 is also critical for licensing of effector cytokine production from all lineages of memory CD4^+^ T cells (25). Indeed, R2^–/–^ mice did show some evidence of increased CD4^+^ T cell number, activation, and IFN-γ expression (Supplemental Figure 6) but no effect on GCs.

Despite the known effects of IL-1 on adaptive responses and the proposed role in the GC (13), littermate WT and global *I1r1*^–/–^ mice show near-identical splenic T cell parameters, GC responses, and anti-sRBC IgG antibody titers (Figure 5). In contrast, Tfr-specific IL-1R2 loss (Figure 3, D–L) or acute global IL-1R2 deficiency (Figure 6) affects both the GC response and T cell parameters. The simplest explanation for this difference is that, under normal conditions in WT mice, IL-1R2 is at a sufficient level to restrain any stimulatory effect of local or systemically derived IL-1. These findings indicate that IL-1 signaling is not obligate for an effective GC response, but rather that additional IL-1 signaling can amplify it. Alternatively, it is conceivable that immune cells somehow compensate for germline *Il1r1* loss, but this would indicate substitution of IL-1R1 signaling by, for example, alternative MyD88 signaling receptors, such as TLRs; however, this seems unlikely. Future work to prove which cells produce and respond to IL-1 (and when) could utilize *Il1a*^fl/fl^, *Il1b*^fl/fl^, and *Il1r1*^fl/fl^ mice, along with Cre drivers for Tfh, Tfr, GC B, and stromal cells. Interestingly, providing a second booster immunization resulted in WT mice producing the same level of anti-sRBC IgG antibodies as those with Tfr-specific IL-1R2 deletion but no additional increase in antibody level in Tfr R2^–/–^ mice after the second immunization (Supplemental Figure 5). A limitation of our study might be that IL-1R2 loss advances the kinetics of a GC response, rather than magnitude, which further study would clarify, along with investigating if absolute number and/or phenotype of Tfr cells changes. Together, increased GC IL-1 signaling can boost antibody titers, but is not obligate and can be phenotypically complemented for by a booster.

Of particular interest and broad relevance to multiple fields using IL-1 family genetic manipulation was the clear phenotypic difference between germline and conditional adult loss of IL-1R2. Thus, germline global loss of IL-1R2 had no effect on GCs or anti-sRBC antibody production and only minor effects on T cells (Supplemental Figure 6). However, global deletion of IL-1R2 in adulthood (Figure 6) fully recapitulated the increased GC and T cell responses that were seen with Tfr-specific IL-1R2 loss (Figure 3, D–L). The most likely explanation for this is that, when IL-1R2 is lost from conception (i.e., germline), some form of compensation during development counteracts for the loss of IL-1R2’s antagonism of IL-1. In contrast, in a fully developed adult immune system, acute loss of IL-1R2 cannot be corrected, and a clear phenotype is seen. Future experiments could test if a defined time point at which this plasticity is lost exists or, indeed, if adult deletion of IL-1R2 is also compensated for over time. This concept is not new, with genetic compensation in yeast reported in 1969, while “genetic robustness” where gene loss is compensated by another with overlapping functions is seen in many model organisms (18). Pragmatically, IL-1RA is functionally most similar to IL-1R2 and also expressed by Tfr cells (13), but is typically expressed in different situations. However, mechanisms underlying compensation are not known. Importantly, previous findings on the involvement of IL-1 family members in various physiological and/or disease processes could clearly be affected by the choice of either germline or conditional genetic models.

Although more research is required, our findings are likely of importance in vaccination. The finding of an IL-1–dependent increase in anti-sRBC IgG antibody titers with Tfr-specific IL-1R2 loss suggests that GC output could be modulated therapeutically, for example, to provide a heightened initial antibody response after initial immunization. This could be achieved by providing more IL-1; however, as stated above, IL-1R2 appears to efficiently block GC IL-1 action in WT mice, suggesting exogenous IL-1 would need to be provided at levels able to outcompete IL-1R2, which could have unwanted inflammatory effects. Alternatively, acutely blocking IL-1R2 antagonism of IL-1 could release the brake and allow endogenous IL-1 to boost GC response. However, immunity has evolved a delicate balance over millions of years, and interfering with this equilibrium could have unexpected consequences, such as loss of tolerance, etc. Interestingly, age and obesity substantially blunt GC reactions (26, 27). Thus, future work could test for correlation between serum IL-1R2 level and age or obesity, and if this in part could explain reduced vaccination efficiency in these populations. Similarly, as serum IL-1R2 increases during infection (28), vaccination in the peri-infection period could blunt the response.

We also show substantially lower serum IL-1R2 in patients with either UC or Crohn’s disease but not vasculitis. Although both have a strong inflammatory component, which could clearly be reduced by IL-1R2, there is also a strong autoimmune component, with autoantibodies to both intestinal and nonintestinal self, and microbial antibodies reported (29). In addition, IL-1R2 has a strong genetic association to inflammatory bowel disease (IBD) in multiple reports (19, 20). Future work could leverage serum/plasma proteome data sets, and the SNPs subsequently predicting a given protein’s serum level, to investigate both association and directionality of serum IL-1R2 level and diseases, including IBD. However, this approach could be confounded by an incorrect assumption that serum IL-1R2 relates to local IL-1R2 generated in, for example, the GC. Alternatively, one could assess IL-1R2 on circulating Tfol-like cells. In addition, IBD is also characterized by a large interpatient heterogeneity, meaning very large data sets are likely required.

In conclusion, in addition to controlling IL-1–driven inflammatory responses, IL-1R2 acts as an important intrinsic brake that limits GC reactions and antibody production after immunization. IL-1R2 is lower in two forms of IBD, but future work will be needed to determine if systemic or cell-associated IL-1R2 levels can predict propensity and/or progression of human disease or if modulation of IL-1R2 function can dictate outcome in, for example, vaccination.

## Methods

### Sex as a biological variable.

As *Foxp3* is X-linked, male mice were used to avoid variance from random X-inactivation and to subsequently allow comparison to the other models.

### Materials.

All materials are from MilliporeSigma unless stated otherwise.

### Animal protocols.

*Il1r2*^fl/fl^ mice were generated by homologous recombination of a targeting vector with F3 and FRT flanked Neo and Puro selection and LoxP sites flanking exon 3 of *Il1r2* into a C57BL/6N Tac ES cells, followed by standard generation of chimeras and Flp removal of selection cassettes (Taconic) (Figure 1A). *Il1r2*^fl/fl^ mice on a C57BL/6J background were crossed to CMV-Cre mice (6054; The Jackson Laboratory) to generate *Il1r2*^+/–^ and *Il1r2*^–/–^ littermates, which were born at expected frequencies with no gross phenotype. *Il1r2*^fl/fl^ mice were crossed to *Foxp3-*Cre^ERT2^ mice (16961; The Jackson Laboratory) to generate *Il1r2*^fl/fl^ ± *Foxp3*-Cre^ERT2^ littermates. *Il1r2*^fl/fl^ mice were also crossed to Rosa26-Cre^ERT2^ mice (4847; The Jackson Laboratory) to generate *Il1r2*^fl/fl^ ± Rosa26*-*Cre^ERT2^ littermates. *Il1r1*^–/–^ mice (30) were also used. Mice were genotyped by PCR with the standard The Jackson Laboratory primers and protocol, except *Il1r1*, which followed ref. 30. Mice were maintained on a 12-hour-light/dark cycle, and normal chow (105, SAFE) and water were available ad libitum. Where indicated, tamoxifen citrate diet (TD.55125; Envigo) was fed to mice for 5 weeks before immunization and continued throughout. With Rosa26-Cre^ERT2^/*Il1r2*^fl/fl^ mice, tamoxifen was delivered i.p. (33 mg/kg/d; in corn oil) for 3 weeks, with a 1-week washout before sRBC injection. All experimental groups received tamoxifen. Anakinra was delivered i.p. (25 mg/kg/d; Sobi) 1 day before sRBC injection and continued throughout. Sheep whole blood (TCS) was washed 4 times (PBS; 1,000*g*, 10 minutes) and enumerated; 2 × 10^9^ sRBCs in PBS were injected via the tail vein, and spleens were phenotyped 8 days later. Where indicated a booster was given 1 week after the first immunization. Euthanasia was performed via a rising concentration of CO_2_. IL-1α (3.3 μg/kg in PBS; Peprotech) was injected into the tail vein and blood sampled via the pedal vein at times indicated. IL-1α (500 ng/kg in PBS) was injected i.p., and the peritoneum was lavaged (PBS) after 6 hours. Bone marrow chimeras were generated by standard methods. Briefly, mice were split-dose irradiated (5.5 gray, 4 hours apart); 10 × 10^6^ bone marrow cells were injected via the tail vein within 2 hours of the final irradiation and left to reconstitute for 4 weeks. Parallel experiments with CD45.1/2 showed approximately 95% donor engraftment, with normal blood counts within 4 weeks. Full blood counts used a scil Vet ABC^+^ (Horiba).

### Spleen immunoprofiling and flow cytometry.

Spleens were sieved (70 μm), before washing (PBS; 350*g*, 5 minutes), resieving (40 μm), RBC lysis (eBioscience), washing, and resuspension in FACS buffer (1% BSA, 0.05% NaN_3_, in PBS) or full RPMI. Cells in FACS buffer were Fc blocked (1:100; Biolegend; 10 minutes, room temperature) before staining for T cell activation with anti-CD4 (1:800; RM4-5; eBioscience), anti-CD8 (1:100; 53-6.7; Biolegend), anti-CD62L (1:80; MEL-14; Biolegend), and anti-CD44 (1:400; IM7; Biolegend; 20 minutes, room temperature); for Tfol cells with anti-CD4, anti-CXCR5 (1:100; L138D7; Biolegend), anti-PD-1 (1:100; 29F.1A12; Biolegend), and anti-IL-1R2 (1:20; 4E2; BD; all 20 minutes, room temperature); for Tregs with anti-CD4, anti-CD25 (1:80; PC61; Biolegend; both 20 minutes, room temperature), before washing, fixation, permeabilization (FOXP3 Fix/Perm; Biolegend) and then anti-Foxp3 (1:20; FJK-16S; Biolegend; 30 minutes, room temperature); for Tfr cells with anti-CD4, anti-CXCR5, anti-PD-1 (1:100; all 20 minutes, room temperature) before washing, fixation, permeabilization and then anti-Foxp3; for GC B cells with anti-B220 (1:100; RA3-6B2; Invitrogen), anti-GL7 (GL7; Biolegend), anti-CD45 (30-F11; Biolegend), anti-CD138 (281-2; Biolegend) (all 1:100; all 20 minutes, room temperature). Where indicated, Tregs/Tfr cells were isolated by MACS (Miltenyi). For intracellular cytokine staining cells, in RPMI were treated with or without ionomycin/PMA/brefeldin A (1:500; Biolegend), incubated (5 hours, 37°C), washed, resuspended in FACS buffer, Fc blocked, stained with anti-CD4 or anti-CD8 (20 minutes, room temperature), washed, fixed, permeabilized (Biolegend), and then incubated with anti–IL-10 (JES5-16E3), anti–IL-17 (TC11-18H10.1), and anti–IFN-γ (XMG1.2) (all 1:100; Biolegend; 30 minutes, room temperature). IL-1R2 on splenic T cells was assessed as per Treg staining above but utilized a single panel of anti-CD3 (145-2C11), anti-CD4, anti-CXCR5, anti-PD1 (RMP1-30), anti-CD25, and anti-Foxp3 (MF-14) (all 1:100; Biolegend) and anti-IL-1R2 (1:20; BD), with parallel staining of IL-1R2^–/–^ splenocytes to validate specificity. To measure anti-sRBC antibodies, sRBCs were washed (PBS; 1,000*g*, 10 minutes), incubated with diluted serum (20 minutes, 4°C), washed, and incubated (30 minutes, 4°C) with goat anti-mouse IgG-488 (A1101; Invitrogen). Peritoneal lavages were fixed in formaldehyde (2%; room temperature, 20 minutes), washed (PBS; 250*g*, 6 minutes), resuspended in FACS buffer at one-fifth of the original volume, and incubated (room temperature, 1 hour) with anti-GR1 (1:200; RB6-8C5; Biolegend). For IL-1R2 on neutrophils and monocytes, whole blood (EDTA) was Fc blocked, incubated (room temperature, 30 minutes) with anti-CD11b (1:800; M1/70; Biolegend), anti-Ly6G (1:80; 1A8; Biolegend), anti-CD115 (1:100; AFS98; eBioscience), and anti-IL-1R2, before RBC lysis. Transfected HeLa cells were incubated (room temperature, 30 minutes) with anti–IL-1R2-PE (1:20; R&D Systems). All samples were washed, resuspended in FACs buffer, and analysis by flow cytometry (Accuri C6 or FACSymphony A3; both BD). Examples of key flow cytometry plots and gating strategies are presented (Supplemental Figures 1, 7, and 8).

### Immunohistochemistry.

Slides were cleared before staining with H&E or antigen retrieval with sodium citrate (10 mM; pH 6), blocking in H_2_O_2_ (3%; 15 minutes), Avidin/Biotin block followed by Superblock (both 10 minutes, room temperature; Vector) and then mouse serum (5%; 1 hour), and incubation (16 hours, 4°C) with anti-GL7 (1:20; Thermo Fisher Scientific) or isotype controls (Abcam). Washed sections were incubated (30 minutes, room temperature) with biotinylated 2ry antibody (1:200) in ChemMate Antibody Diluent (Dako) and then ABComplex (Vector; 30 minutes, room temperature), before visualization with DAB (Vector).

### ELISA.

Serum IL-1R2 was measured by ELISA for mouse (capture antibody [1 μg/mL in PBS; AF-563; R&D Systems], detection antibody [67 ng/mL in 0.1% BSA, 0.05% Tween-20 in PBS; BAF-563; R&D Systems] or human [Quantikine; R&D Systems], and human IL-1α in mouse serum was also measured by ELISA [DuoSet; R&D Systems]), all after a 1:3 dilution, following the manufacturers’ instructions, with absorbance measured on a plate reader (BMG Labtech).

### Human samples.

Patients 18 years or older with active ANCA-associate AAV on no or minimal treatment were recruited from the Lupus and Vasculitis Service at Addenbrooke’s Hospital. Active disease was defined by the Birmingham Vasculitis Activity Score. Patients with active IBD (Crohn’s disease or UC), were recruited from the IBD Clinic at Addenbrooke’s Hospital, before commencing treatment. Diagnosis was based on standard endoscopic and histological and radiological criteria, and it was confirmed by ≥1 objective marker (e.g., raised CRP, calprotectin). Age-matched individuals acting as healthy controls were recruited from the National Institute for Health Research BioResource.

### Statistics.

Data are presented as mean ± SEM, unless otherwise stated. All statistical analyses were carried out using Prism 7 (Graphpad). All assays that produced continuous data, with the exception of flow cytometry and mouse experiments, were performed in duplicate. *n* = an individual experimental replicate performed on a different day or an individual mouse — never a technical replicate. Before statistical testing for significance, data were analyzed for normality with a Shapiro-Wilks test, with normal distribution analyzed by parametric and nonnormal by nonparametric. Parametric test analysis of continuous data used unpaired *t* test (2-tailed) or 2-way ANOVA with Dunnett’s post hoc or Tukey’s post hoc multiple comparisons test. Nonparametric tests used the Mann-Whitney *U* test or the Kruskal-Wallis test. A *P* value of less than 0.05 was considered significant.

### Study approval.

Animal protocols were performed under UK Home Office licensing (London, United Kingdom). For human studies, ethical approval was provided by the Cambridgeshire Regional Ethics Committee (Cambridge, United Kingdom) (08/H0306/21 and 08/H0308/176), and all individuals provided written informed consent.

### Data availability.

Data and supporting information are available from MCHC upon reasonable request. Values for all data points in graphs are reported in the Supporting Data Values file.

## Author contributions

KP, MH, LAK, and AR performed and designed experiments and analyzed data. KGCS and PAL provided patient samples. PAL, MN, MRB, and ZM provided helpful discussion. MCHC conceived the project, performed and designed experiments, analyzed data, and wrote the manuscript.

## Supplementary Material

Supplemental data

Unedited blot and gel images

Supporting data values

## Version 1 02/08/2024

In-Press Preview

## Version 2 03/08/2024

Electronic publication
