# Supplementary material for: Loss of T follicular regulatory cell–derived IL-1R2 augments germinal center reactions via increased IL-1
Source: JCI Insight. 2024 Feb 8;9(5):e174005. doi: 10.1172/jci.insight.174005 (PMC11143922; doi:10.1172/jci.insight.174005)
Supplement: Supplemental data [file jciinsight-9-174005-s030.pdf]

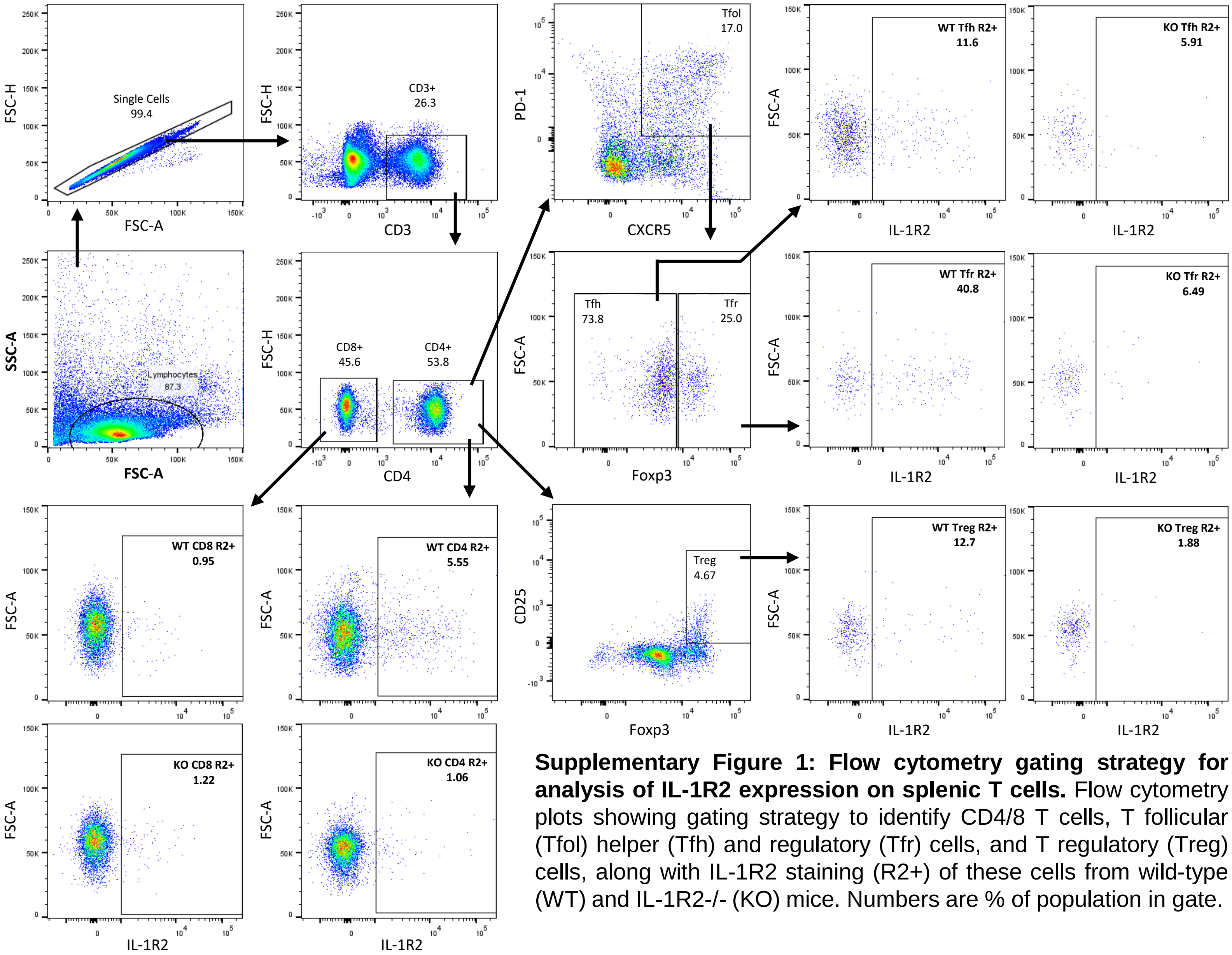

Supplementary Figure 1: Flow cytometry gating strategy for analysis of IL-1R2 expression on splenic T cells. Flow cytometry plots showing gating strategy to identify CD4/8 T cells, T follicular (Tfol) helper (Tfh) and regulatory (Tfr) cells, and T regulatory (Treg) cells, along with IL-1R2 staining (R2+) of these cells from wild-type (WT) and IL-1R2-/- (KO) mice. Numbers are % of population in gate.

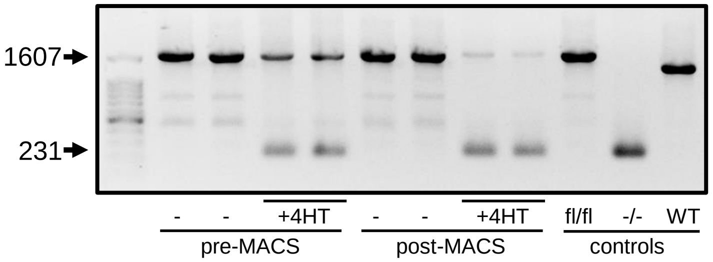

Supplementary Figure 2: *Il1r2*$^{fl/fl}$/*Foxp3*-Cre-ER^{T2} mice show Tfr-specific *Il1r2* deletion after tamoxifen treatment. RT-PCR for an *Il1r2* amplicon in splenic cDNA from *Il1r2*$^{fl/fl}$/*Foxp3*-Cre-ER^{T2} mice treated ±tamoxifen (+4HT), before or after MACS sorting for Treg/Tfr. Control lanes are genomic DNA from *Il1r2* flox/flox (fl/fl), *Il1r2*-/- (-/-) and *Il1r2*+/+ (WT) mice. Data represents mean ±SEM, n=2 mice per ±4HT group, with spleens divided for ±MACS sorting.

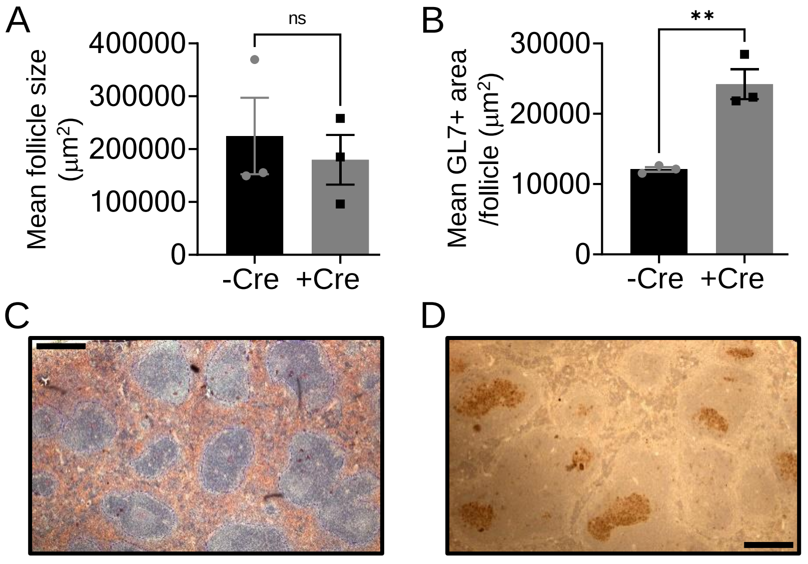

Supplementary Figure 3: GC follicle size and gross morphology is normal, but GL7+ cells are increased with loss of Tfr IL-1R2. *Il1r2*$^{fl/fl}$ ±Foxp3-Cre-ER^{T2} (Cre) littermate mice were all tamoxifen treated, immunised with sheep red blood cells (sRBC) and spleens fixed, processed and sectioned 8 d later. **(A,B)** Mean follicle size **(A)** and GL7+ area per follicle **(B)** in spleens from genotypes as indicated. **(C,D)** Example H&E staining and quantification of follicle size **(C)**, and example immunohistochemistry for GL7 **(D)**. Data represents mean ±SEM; n=3/3 individual mice, with counting of multiple follicles per mouse. p = **≤0.01; ns = not significant, using T test. Scale bar = 500μm.

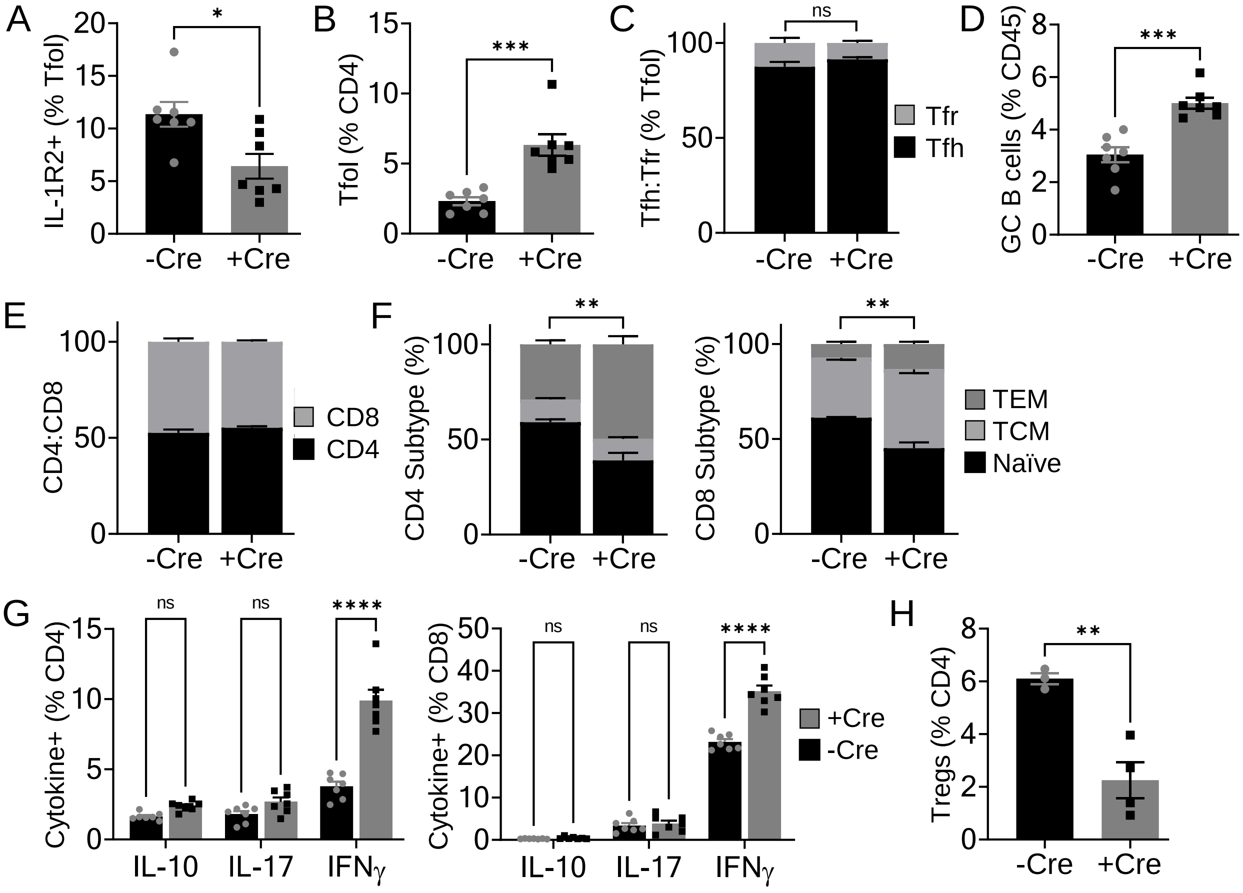

Supplementary Figure 4: Ova/Alum immunisation also increases the GC response in mice with IL-1R2 deficient Tfregs. *Il1r2*$^{fl/fl}$ ±Foxp3-Cre-ER^{T2} (Cre) littermate mice were all tamoxifen treated, immunised with Ova/Alum and spleens immunophenotyped 8 d later. **(A-D)** Flow cytometry for IL-1R2 on splenic T follicular cells (Tfol) **(A)**, Tfol cells **(B)**, ratio of Tfh to Tfr cells **(C)** and germinal centre (GC) B cells **(D)** in the genotypes indicated. **(E,F)** Flow cytometry for splenic CD4/8 T cell ratio **(E)** and CD4/8 T cell subtype **(F)** in the genotypes indicated. TCM = central memory, TEM = effector memory. **(G)** Intracellular cytokine staining in splenic CD4/8 T cells activated with PMA/Ionomycin. **(H)** Flow cytometry for splenic Tregs. Data represents mean ±SEM; n=7/7 individual mice and representative of ≥2 repeats. p = *≤0.05, **≤0.01, ***≤0.001, ****≤0.0001; ns = not significant, using T test and ANOVA.

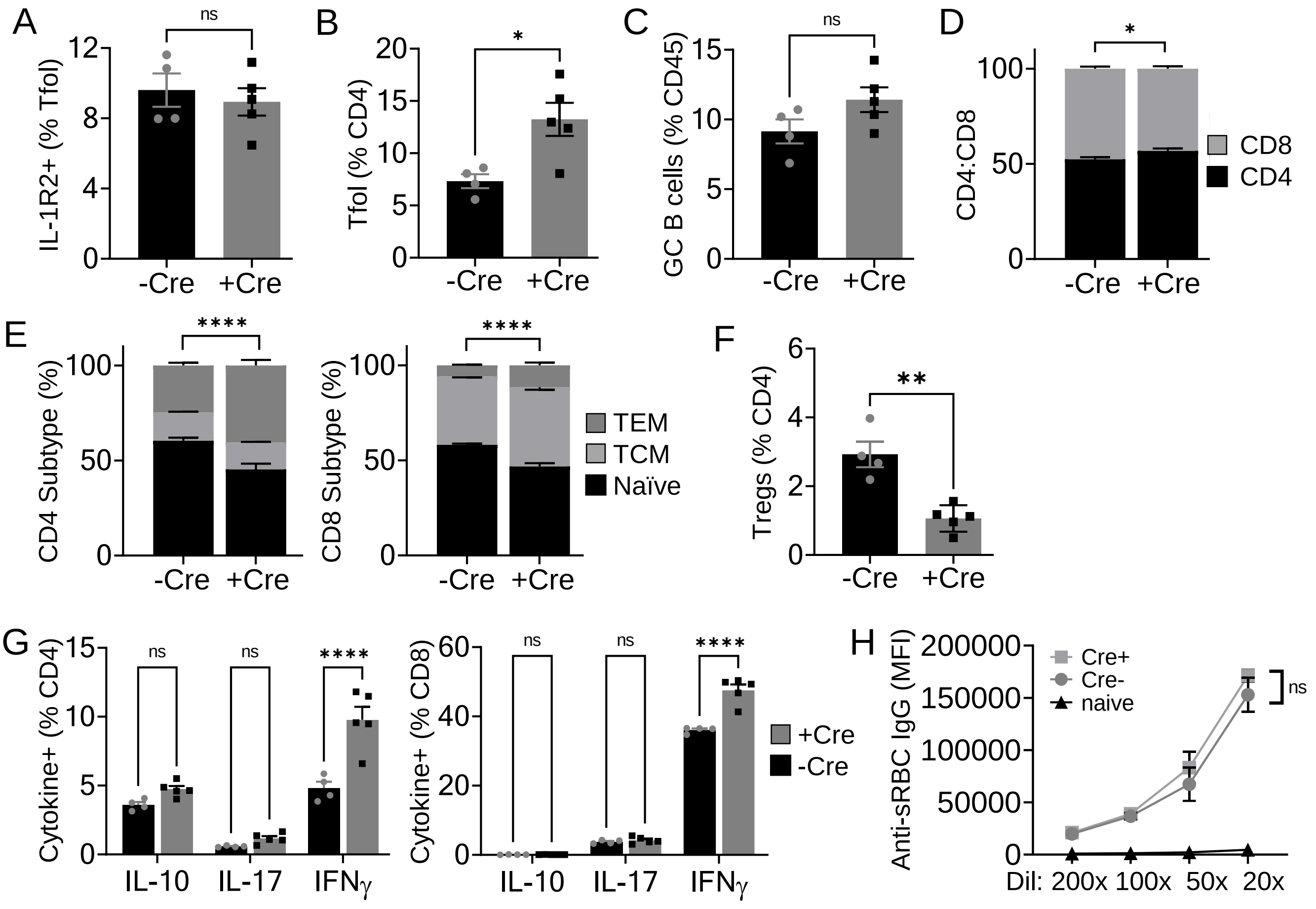

Supplementary Figure 5: Tfr IL-1R2 deficiency does not increase GC responses after a booster immunisation. *Il1r2*$^{fl/fl}$ ±Foxp3-Cre-ER^{T2} (Cre) littermate mice were all tamoxifen treated, immunised twice with sheep red blood cells (sRBC) and spleens immunophenotyped 8 d later. **(A-C)** Flow cytometry for IL-1R2 on splenic T follicular cells (Tfol) **(A)**, Tfol cells **(B)** and germinal centre (GC) B cells **(C)** in the genotypes indicated. **(D-F)** Flow cytometry for splenic CD4/8 T cell ratio **(D)**, CD4/8 T cell subtype **(E)** and Tregs **(F)** in the genotypes indicated. TCM = central memory, TEM = effector memory. **(G)** Intracellular cytokine staining in splenic CD4/8 T cells activated with PMA/Ionomycin. **(H)** Flow cytometry for binding of serum anti-sRBC IgG antibodies to sRBCs. Data represents mean ±SEM; n=4/5 individual mice and representative of ≥2 repeats. p = *≤0.05, **≤0.01, ****≤0.0001; ns = not significant, using T test and ANOVA.

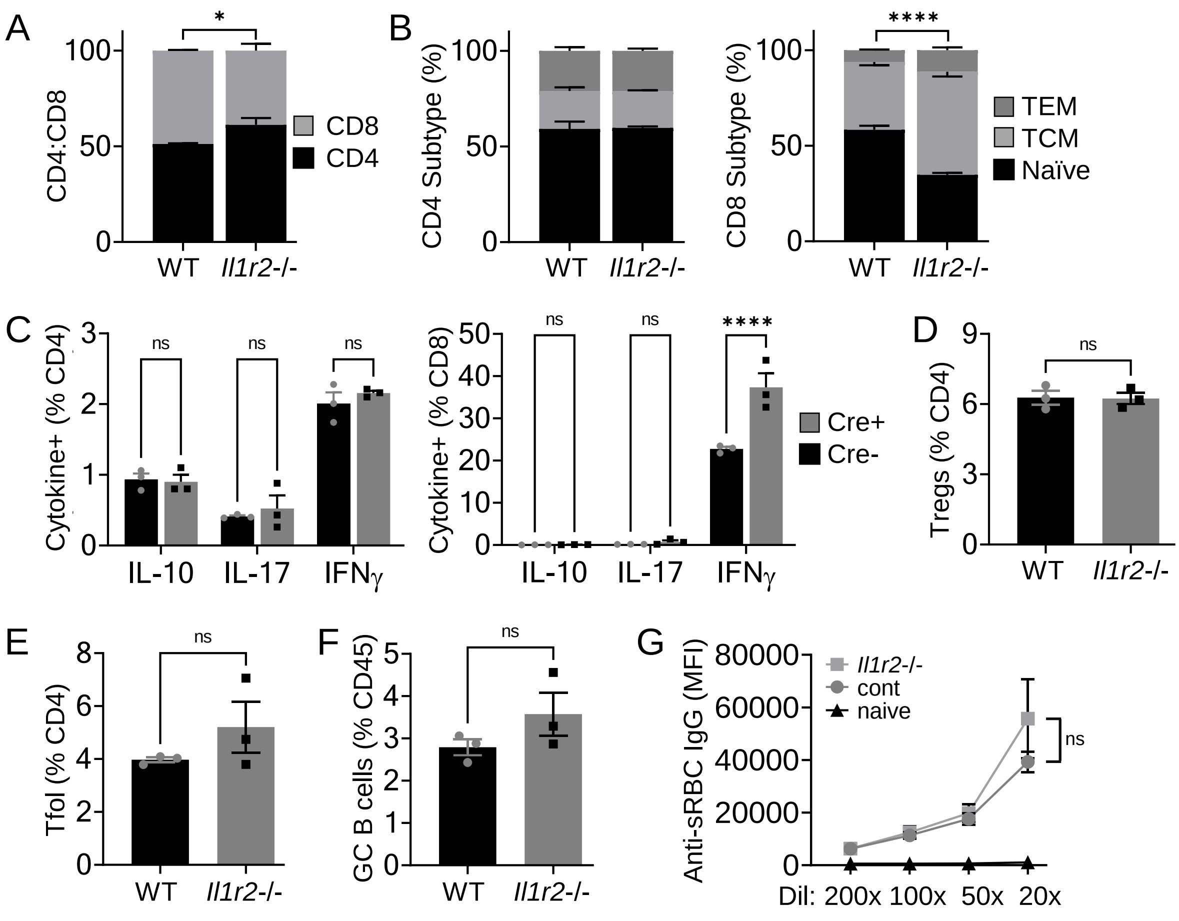

Supplementary Figure 6: Global loss of IL-1R2 alters T cell function after immunisation. *Il1r2-/-* and *Il1r2*+/+ (WT) littermate mice were immunised with sheep red blood cells (sRBC) and spleens immunophenotyped 8 d later. **(A,B)** Flow cytometry for splenic CD4/8 T cell ratio **(A)** and CD4/8 T cell subtype **(B)** in the genotypes indicated. TCM = central memory, TEM = effector memory. **(C)** Intracellular cytokine staining in splenic CD4/8 T cells activated with PMA/Ionomycin. **(D-F)** Flow cytometry for splenic Tregs. T follicular cells (Tfol) **(E)** and germinal centre (GC) B cells **(F)** in the genotypes indicated. **(G)** Flow cytometry for binding of serum anti-sRBC IgG antibodies to sRBCs. Dil = serum dilution. Data represents mean ±SEM, n=3/3 individual mice, and representative of ≥2 repeats. p = *≤0.05, ****≤0.0001; ns = not significant, using T test and ANOVA.

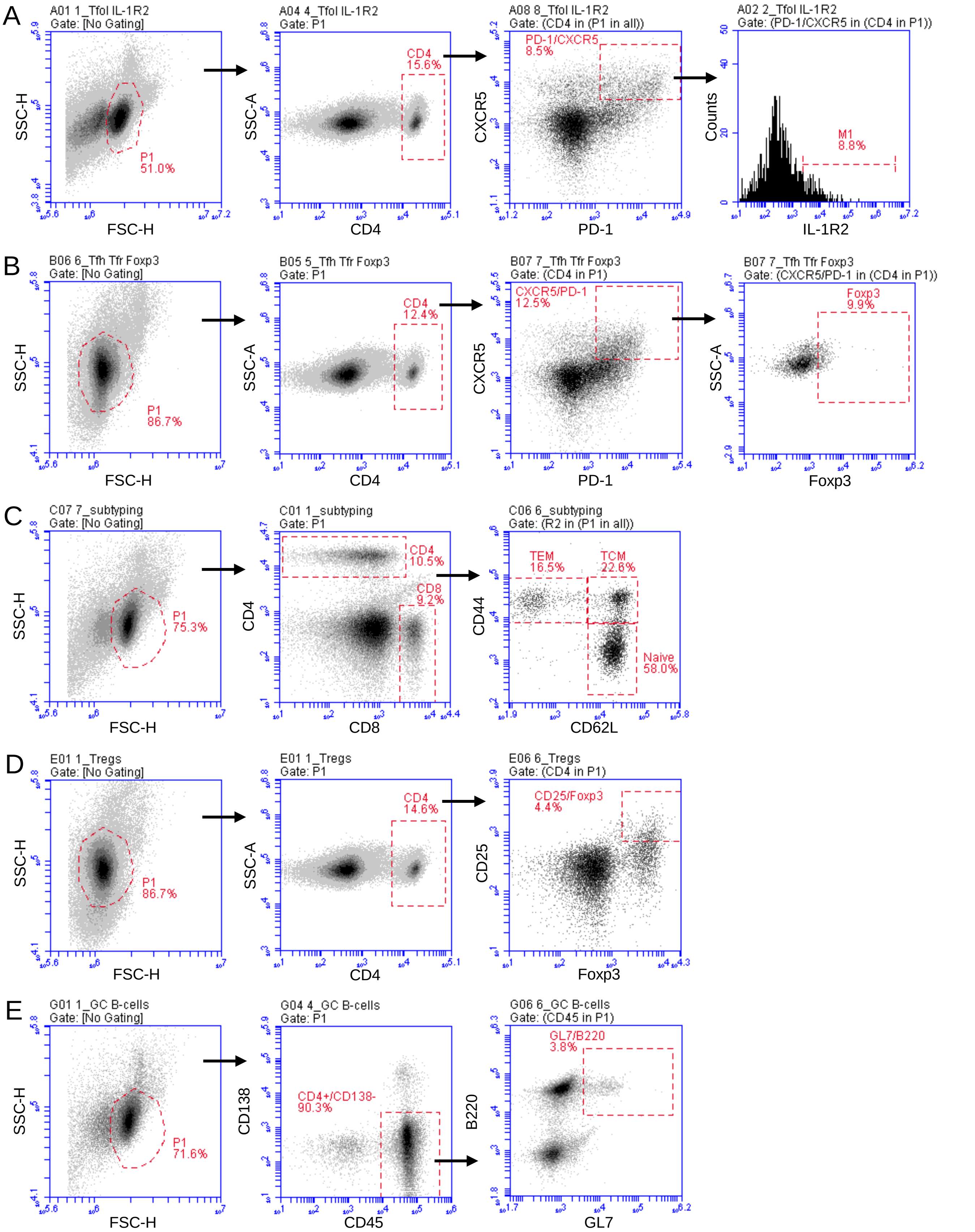

Supplementary Figure 7: Flow cytometry plots and example gating strategies for spleen immunophenotyping. (A-E) Spleen immunophenotyping by flow cytometry for T follicular cell IL-1R2 expression **(A)**, T follicular regulatory cells **(B)**, CD4/8 T cell subtype **(C)**, Tregs **(D)**, and germinal centre (GC) B cells **(E)**. TCM = central memory, TEM = effector memory.

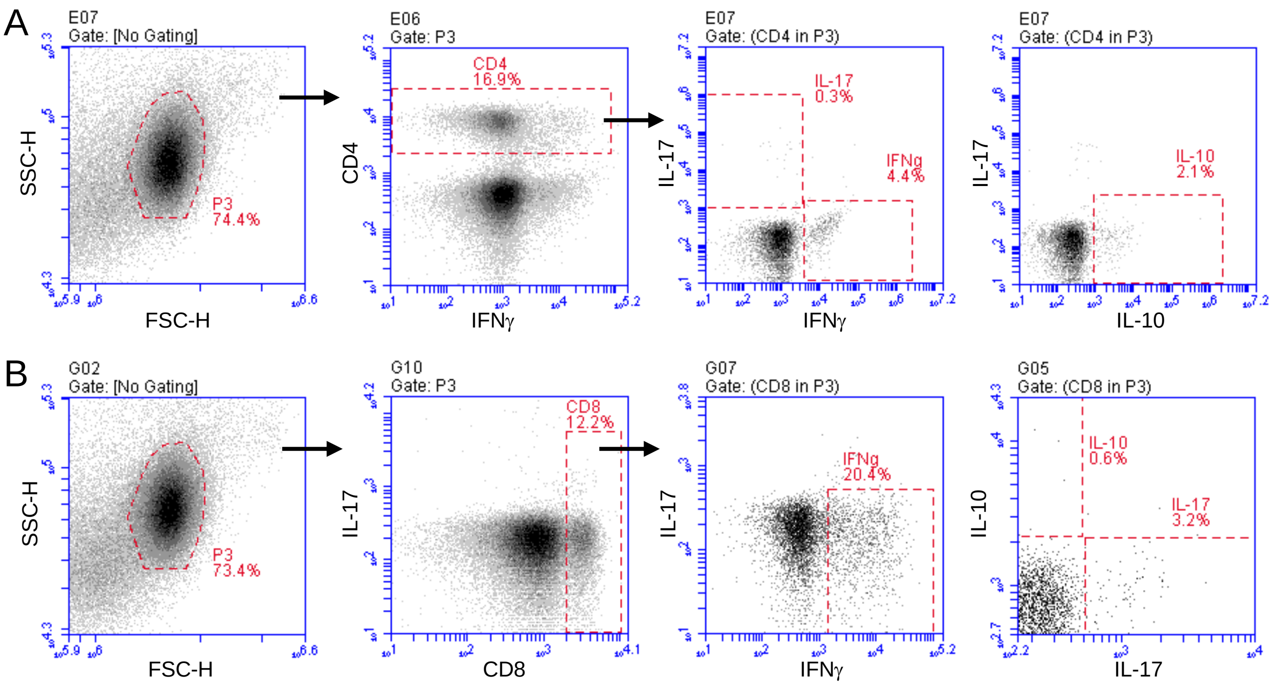

Supplementary Figure 8: Flow cytometry plots and example gating strategies for splenic T cell cytokine content. (A,B) Intracellular cytokine staining by flow cytometry for interferon gamma (IFNγ), IL-10 and IL-17 in CD4 **(A)** and CD8 **(B)** T cells treated with ionomycin, PMA and brefeldin A.
