# Supplementary figures and images for: Loss of T follicular regulatory cell–derived IL-1R2 augments germinal center reactions via increased IL-1
Source: JCI Insight. 2024 Feb 8;9(5):e174005. doi: 10.1172/jci.insight.174005 (PMC11143922; doi:10.1172/jci.insight.174005)

## Slide 1
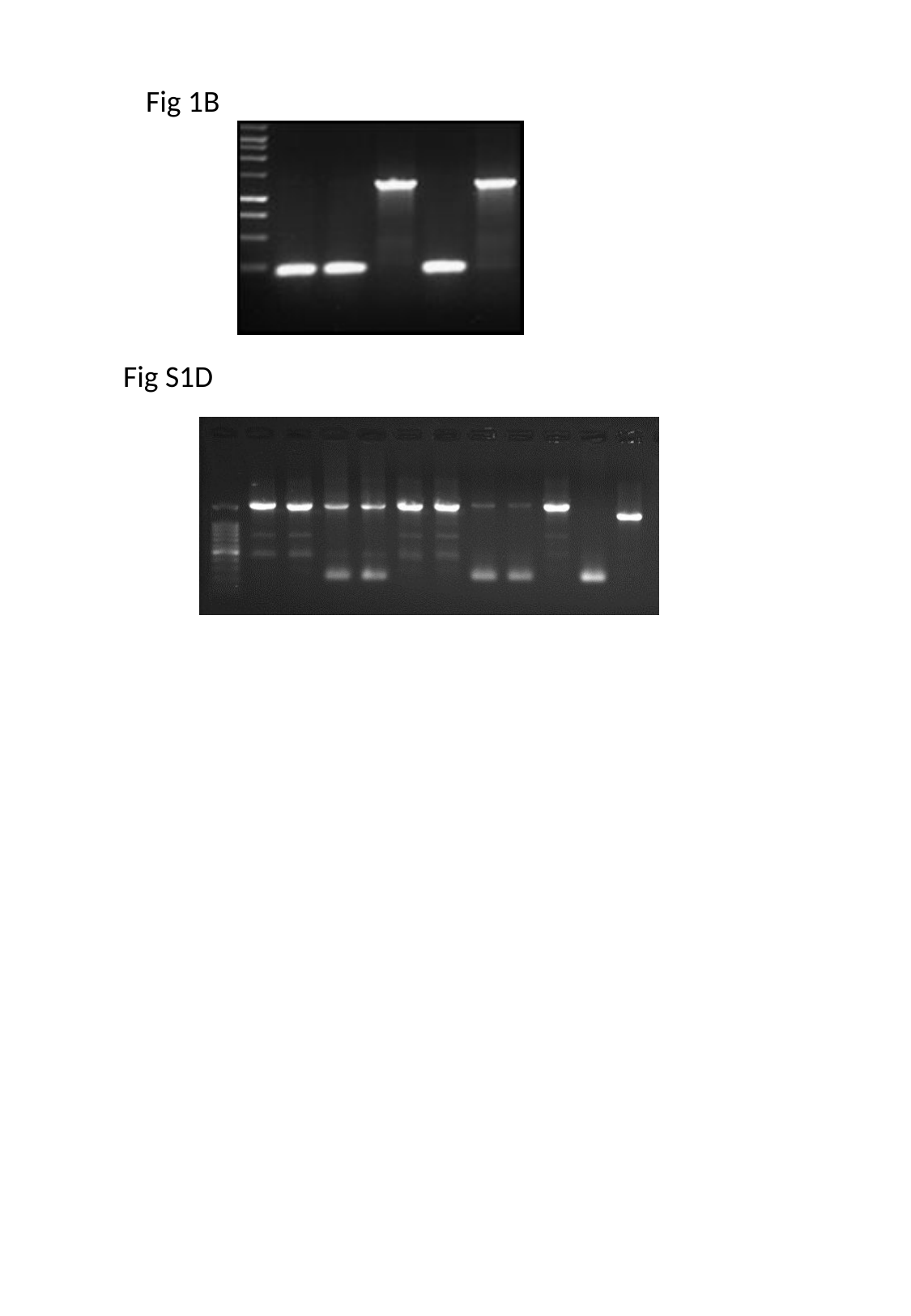

Fig 1B
Fig S1D

Supplement: Unedited blot and gel images [file jciinsight-9-174005-s031.pptx]
